# Effects of etizolam and ethyl loflazepate on the P300 event-related potential in healthy subjects

**DOI:** 10.1186/1744-859X-9-37

**Published:** 2010-11-03

**Authors:** Goro Fukami, Tasuku Hashimoto, Yukihiko Shirayama, Tadashi Hasegawa, Hiroyuki Watanabe, Mihisa Fujisaki, Kenji Hashimoto, Masaomi Iyo

**Affiliations:** 1 Department of Psychiatry, Chiba University Graduate School of Medicine, Chiba, Japan; 2 Division of Clinical Neuroscience, Chiba University Center for Forensic Mental Health, Chiba, Japan

## Abstract

**Background:**

Benzodiazepines carry the risk of inducing cognitive impairments, which may go unnoticed while profoundly disturbing social activity. Furthermore, these impairments are partly associated with the elimination half-life (EH) of the substance from the body. The object of the present study was to examine the effects of etizolam and ethyl loflazepate, with EHs of 6 h and 122 h, respectively, on information processing in healthy subjects.

**Methods:**

Healthy people were administered etizolam and ethyl loflazepate acutely and subchronically (14 days). The auditory P300 event-related potential and the neuropsychological batteries described below were employed to assess the effects of drugs on cognition. The P300 event-related potential was recorded before and after drug treatments. The digit symbol test, trail making test, digit span test and verbal paired associates test were administered to examine mental slowing and memory functioning.

**Results:**

Acute administration of drugs caused prolongation in P300 latency and reduction in P300 amplitude. Etizolam caused a statistically significant prolongation in P300 latency compared to ethyl loflazepate. Furthermore, subchronic administration of etizolam, but not ethyl loflazepate, still caused a weak prolongation in P300 latency. In contrast, neuropsychological tests showed no difference.

**Conclusions:**

The results indicate that acute administration of ethyl loflazepate induces less effect on P300 latency than etizolam.

## Background

Benzodiazepines have anxiolytic, sedative, anticonvulsant and myorelaxant properties, and have been widely prescribed in various clinical settings. These compounds, however, also induce adverse effects such as oversedation, cognitive impairment, motor impairment and withdrawal. These adverse effects may be partly associated with the elimination half-life (EH) of the compounds from the body; that is, long-term use of the compounds with a short elimination rate may induce withdrawal syndromes, whereas accumulation-related effects of a long elimination rate may include oversedation, cognitive dysfunction and motor impairment [1-4].

It has been observed previously that cognitive impairment induced by benzodiazepines may go unnoticed while profoundly disturbing social activity [5]. Therefore, it is clinically very important to take note of the cognitive effects of benzodiazepines. In order to assess the effects of benzodiazepines on cognition, the event-related potential (ERP), P300, may be useful [6], as well as neuropsychological tests. The P300 components of ERP are elicited by an auditory oddball paradigm in which a subject detects infrequent task-relevant stimuli randomly presented among frequent stimuli. P300 reflects stimulus context and stimulus meaning [7]. P300 components are associated with cognitive processes such as attention, memory, orientation and evaluation. Relationships between P300 and neuropsychological function have been reported [8-14]. Benzodiazepine anxiolytic drugs, as well as benzodiazepine hypnotic drugs, have been reported to induce reductions in P300 amplitude and prolongation in P300 latency [6,15-20].

As far as is known, however, there are no reports on the effects of chronic or subchronic administration of benzodiazepines on cognition and P300 from the viewpoint of elimination rates. Here, we studied the effects of anxiolytic benzodiazepines on neuropsychological functions and P300 components of auditory ERP under acute and subchronic administration of ethyl loflazepate and etizolam. Ethyl loflazepate is a potent, non-sedative, anxiolytic drug with a long EH of 122 h [21], whereas etizolam is characteristic of a potent antianxiety and sedative drug with a short EH of 6 h [22]. Therefore, etizolam is often used as a sleep inducer. However, it is well known that benzodiazepine drugs including etizolam and ethyl loflazepate have the effects of reducing the deep sleep stage 3, resulting in loss of good sleep. The aim of the present study is to examine whether sedative or anxiolytic actions of benzodiazepines have some effects on ERP and neuropsychological tests.

## Methods

### Study design and subjects

All subjects had normal acoustic function and were right handed. The ethics committee of Chiba University Graduate School of Medicine approved the experiments. Subjects were free from treatment for past psychiatric illness. Written informed consent was obtained after the procedure had been fully explained.

In the acute experiment, 10 healthy men (n = 5) and women (n = 5) ranging in age from 16 to 38 (average age 28.6 (SD 6.5)) participated in the study. First, all subjects were measured for the P300 components of ERP and received neuropsychological tests. Then they took etizolam (1 or 2 mg, orally). Then, 2 h later, the same ERP and neuropsychological tests were performed, since the blood concentration of the drugs reaches a maximum 1-2 h after consumption. After a 2-week washout period, the same experimental procedures were repeated, but subjects took ethyl loflazepate (1 or 2 mg, orally).

In the subchronic experiment, 17 healthy men (n = 8) and women (n = 9) ranging in age from 22 to 34 (average age 27.4 (SD 4.1)) participated in the study. The 17 subjects were divided into 2 groups: the first group was given etizolam (1 mg, orally, for 14 days), and the second given ethyl loflazepate (1 mg, orally for 14 days). Subjects were asked to take drugs in the evening every day, and performed ERP recording and neuropsychological tests 14-20 h after taking the last drug. Subjects performed ERP recording and neuropsychological tests twice before and after subchronic treatment with etizolam or ethyl loflazepate.

Doses examined in the present study were chosen based on the equivalent conversion table for anxiolytic drugs (5 mg of diazepam, 1.5 mg of etizolam, and 1.67 mg of ethyl loflazepate) [23,24].

### ERP procedure

Electroencephalogram electrodes were attached at Fz, Cz and Pz according to the international 10-20 system. Earlobe electrodes were linked for reference. Electro-oculography was also recorded from vertical and lateral derivations to check ocular artefacts. Subjects sat on a semi-reclined chair in a sound-attenuated and electrically shielded room during recordings. Subjects were instructed to press a button as quickly as possible upon hearing the infrequent high-pitched tones. Event-related potentials were recorded under an oddball paradigm. The stimuli consisted of a 1,000 Hz tone burst (frequent non-target stimulus) and a 2,000 Hz tone burst (rare target stimulus). In each paradigm, 200 stimuli were presented through bilateral earphones by using a Neuropack 10 (MEB-2200, Nihon Kohden, Tokyo, Japan). The ratio of the rare versus frequent stimuli was 0.25. Stimuli were presented in a random order, the duration of each stimulus being 120 ms, with rise and fall times of 10 ms. The intensity was 40 dB for all stimuli. The interstimulus interval was 1.5 s.

### Neuropsychological tests

The trail making test consists of two parts [25]. In part A, subjects are asked to draw lines connecting 25 consecutively numbered circles on a worksheet. In part B, they draw lines connecting 25 consecutively numbered and lettered circles, alternating between the sequences (for example, 1-A-2-B-3 and so on). Part A examines psychomotor speed and attention. Part B examines set alternation or divided attention.

The digit symbol modalities test is a measure of switching attention [26]. Subjects are asked to identify nine different symbols corresponding to the numbers 1 through 9, and write the correct number under the corresponding symbol. Thus, visual shifting and pairing of specific digits is directed, with a set of prespecified symbols.

The forward digit span test is a measure of simple attention, immediate memory and attentional control processing. In contrast, backward digit span is not only a test of attentional control processing but also working memory test.

The verbal paired associates test from the Wechsler Memory Scale-Revised (WMS-R) is a cued recall test of verbal memory [27]. Subjects learned a list of eight verbal paired associates. Then, either immediately or after a delay, the examiner says one word of each pair and the subjects recall the other word. Three sets of immediate memory testing and one set of delayed recall testing were administered.

### Statistical analysis

Two-way repeated measures analysis of variance (ANOVA) was performed to assess the overall differences between variables. Where a significant interaction in the within-subject variables was found, subsequent one-way ANOVA was carried out among more than three groups by a *post hoc *comparison using Fisher's protected least significant difference test. For comparison of the mean values between the two groups, statistical evaluation was performed using the two-tailed Student's t test. The significance level was set at *P *< 0.05.

## Results

### Effects of acute treatments with etizolam and ethyl loflazepate on P300

**Figure 1:**
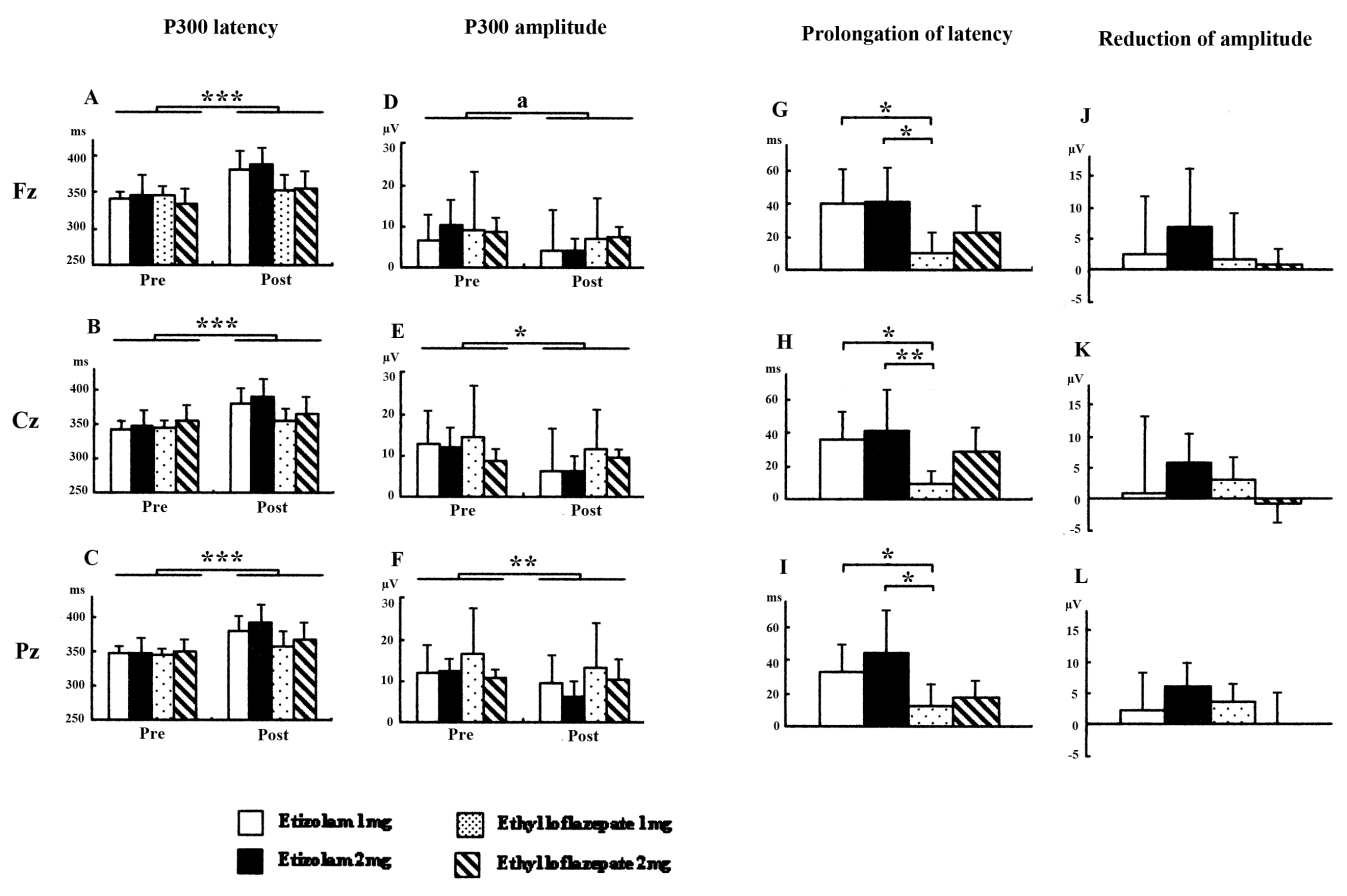
**Effects of acute treatments with etizolam and ethyl loflazepate on P300**. *P < 0.05, **P < 0.01, ***P < 0.001 compared to pretreatment (repeated analysis of variance (ANOVA)). **(a) **Trend for changes without significance. *P < 0.05, **P < 0.01 compared to ethyl loflazepate with low dose (1 mg) (ANOVA followed by Fisher's protected least significant difference test).

For acute drug treatment on the P300, two-way repeated ANOVA indicated significant effects of treatment (Fz, F (1,16) = 49.397, *P *< 0.0001; Cz, F (1,16) = 59.022, *P *< 0.0001; Pz, F (1,16) = 45.623, *P *< 0.0001), but not effects of group, on latency, with a significant interaction (Fz, treatment × group, F (3,16) = 3.846, *P *= 0.0301; Cz, treatment × group, F (3,16) = 3.436, *P *= 0.0423; Pz, treatment × group, F (3,16) = 3.278, *P *= 0.0483) (Figure 1a-c). The subsequent one-way ANOVA on the changes of P300 latency indicated significant differences (Fz, F (3,16) = 3.431, *P *= 0.0425; Cz, F (3,16) = 3.436, *P *= 0.0423; Pz, F (3,16) = 3.387, *P *= 0.0441), and the *post hoc *comparison using Fisher's protected least significant difference test indicated the following: ethyl loflazepate 1 mg has less effects than etizolam 1 mg and etizolam 2 mg in Fz (Figure 1g), Cz (Figure 1h), and Pz (Figure 1i). For amplitude, two-way repeated ANOVA indicated significant effects of treatment (Cz, F (1,16) = 7.967, *P *= 0.0123; Pz, F (1,16) = 8.807, *P *= 0.0091; but see Fz, F (1,16) = 4.032, *P *= 0.0618), but not effects of group, without a significant interaction (Figure 1d-f). This seems to reflect that benzodiazepine reduced P300 amplitude. Although the subsequent one-way ANOVA on the changes of P300 amplitude revealed no significant difference among drug groups (Figure 1j-l), the magnitude of changes showed that etizolam (2 mg) produced a trend in reduction of amplitude.

### Effects of subchronic treatments with etizolam and ethyl loflazepate on P300

**Figure 2:**
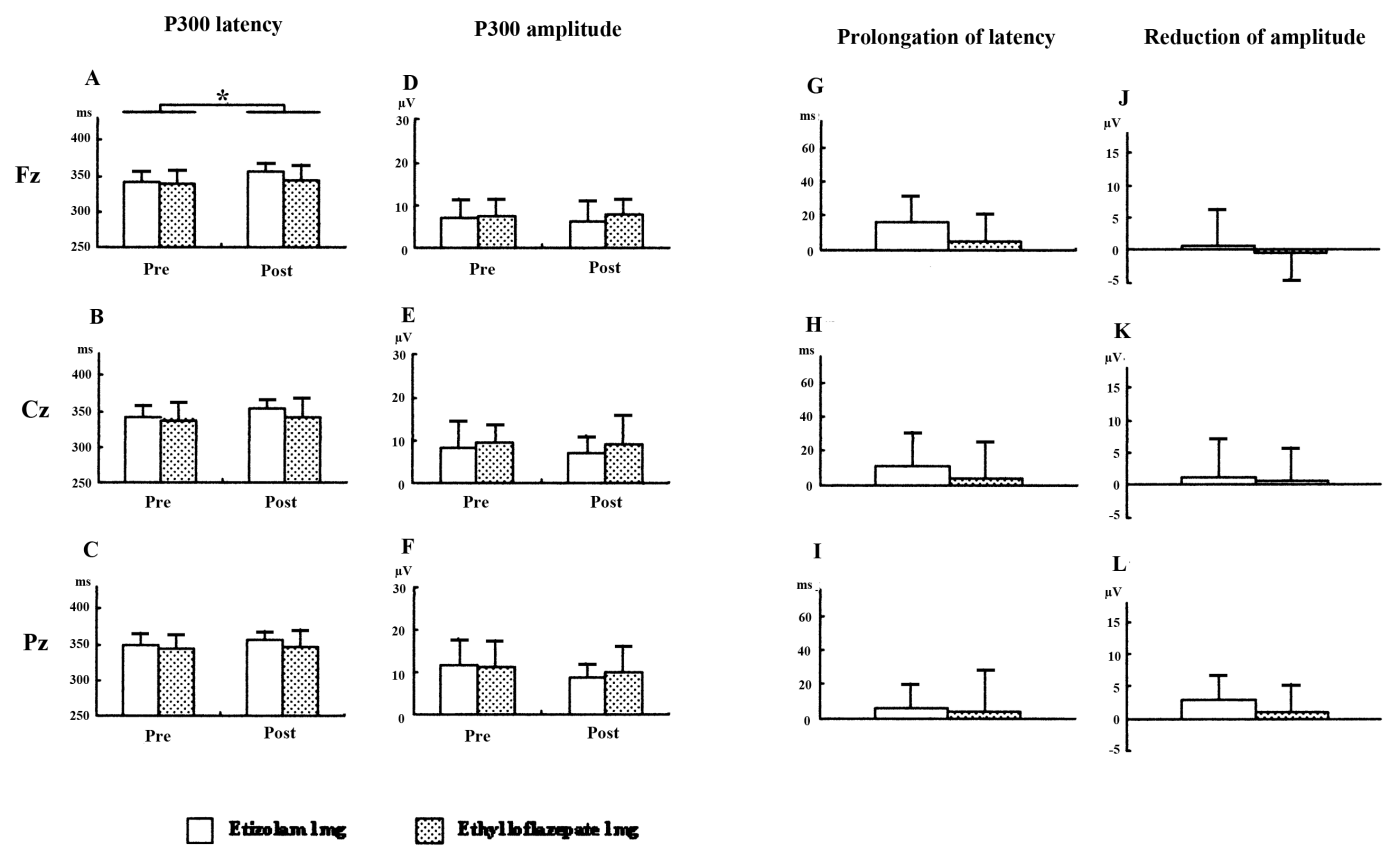
**Effects of subchronic treatments with etizolam and ethyl loflazepate on P300**. *P < 0.05 compared to pretreatment (repeated analysis of variance (ANOVA)).

For subchronic drug treatment on the P300, two-way repeated ANOVA indicated significant effects of treatment region specifically (Fz, F (1,15) = 7.734, *P *= 0.0140; but see Cz, F (1,15) = 2.391, *P *= 0.1491; Pz, F (1,15) = 0.954, *P *= 0.3443), but not effects of group, on latency, without a significant interaction (Figure 2a-c). The subsequent Student t test on the changes of P300 latency revealed no significant difference between two drugs (Figure 2g-i). With regard to amplitude, two-way repeated ANOVA indicated no significant effects of treatment without a significant interaction (Figure 2d-f, j-l).

### Effects of treatments with etizolam and ethyl loflazepate on neuropsychological tests

**Figure 3:**
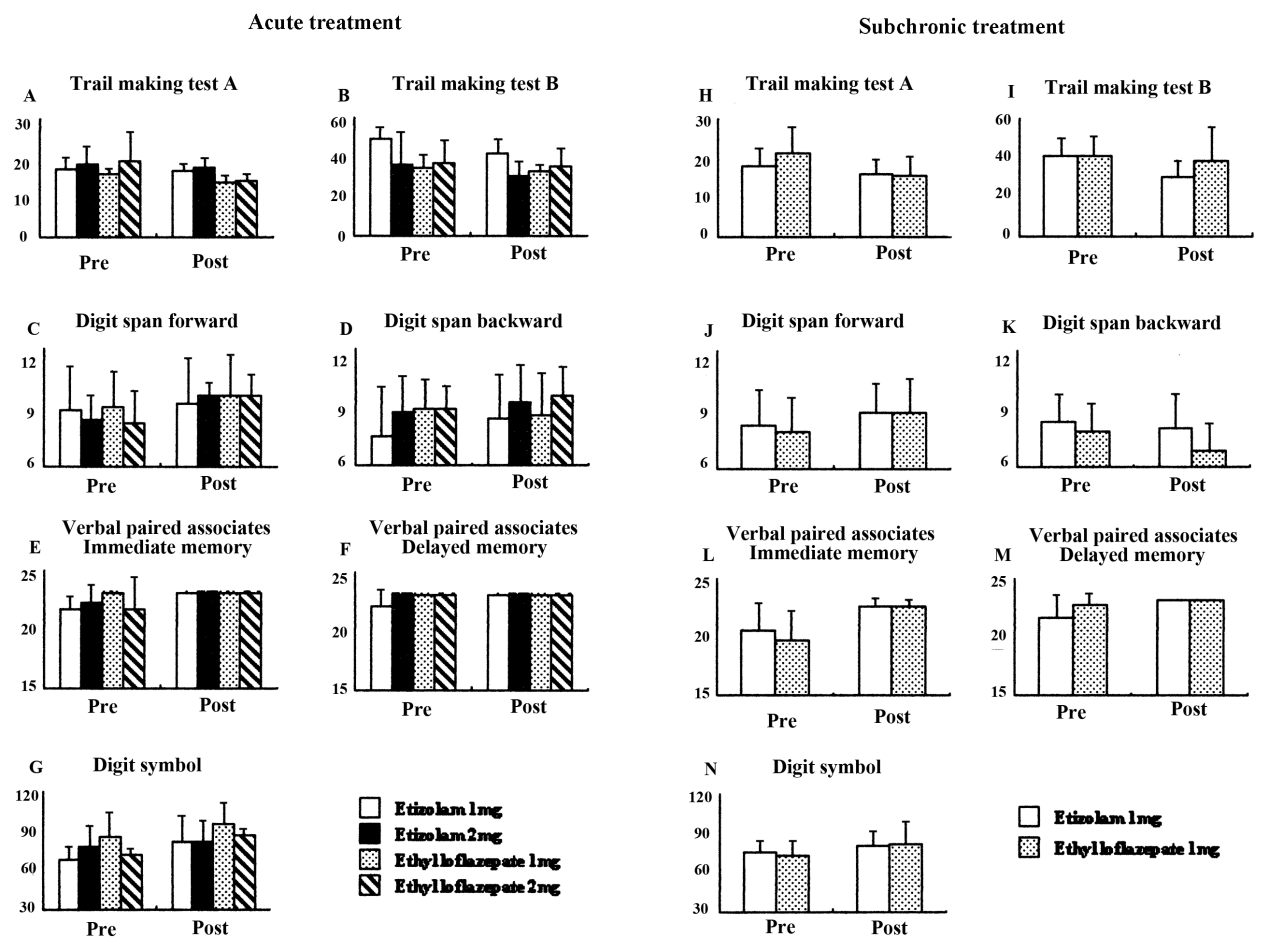
**Effects of acute and subchronic treatment with etizolam and ethyl loflazepate on neuropsychological tests**.

For acute effects of drug treatment on neuropsychological tests, two-way repeated ANOVA showed significant practice effects of repeated testing, but not effects of drug group, on test scoring without a significant interaction in some tests including trail making A (F (1,16) = 7.399, *P *= 0.0151, Figure 3a), trail making B (F (1,16) = 8.409, *P *= 0.0104, Figure 3b), digit span forward (F (1,16) = 8.696, *P *= 0.0094, Figure 3c), verbal paired associates immediate memory (F(1,16) = 6.485, *P *= 0.0215, Figure 3e) and digit symbol (F(1,16) = 24.209, *P *= 0.0002, Figure 3g), and no significant effects of repeated testing and drugs on test scoring without a significant interaction in other tests such as digit span backward (Figure 3d) and verbal paired associates delayed recall (Figure 3f).

For subchronic effects of drug treatment on neuropsychological tests, two-way repeated ANOVA indicated significant practice effects of repeated testing, but not effects of drug group, on test scoring without a significant interaction, in some tests including trail making test A (F (1,15) = 12.472, *P *= 0.0030, Figure 3h), trail making test B (F (1,15) = 5.426, *P *= 0.0342, Figure 3i), digit span forward (F (1,15) = 7.092, *P *= 0.0177, Figure 3j), verbal paired associates immediate memory (F (1,15) = 16.449, *P *= 0.0010, Figure 3l), verbal paired associates delayed recall (F (1,15) = 5.773, *P *= 0.0297, Figure 3m) and digit symbol (F (1,15) = 6.075, *P *= 0.0236, Figure 3n), and no significant effects of repeated testing and drug group on test scoring without a significant interaction in digit span backward test (Figure 3k).

## Discussion

Our results show acute drug treatment induced prolongation in P300 latency. This is consistent with previous studies demonstrating that benzodiazepines such as alprazolam, lorazepam, clonazepam and triazolam induce prolongation in P300 latency [6,16,18,19]. However, subsequent ANOVA revealed that etizolam (1 and 2 mg) induced significant prolongation in P300 latency compared to ethyl loflazepate (1 mg). The difference between the acute effects of etizolam and ethyl loflazepate could contribute to the potent sedative effects of etizolam, although equivalent doses of these two drugs to diazepam are clinically almost the same. P300 latency is suggested to reflect the stimulus evaluation time, and is relatively independent of response selection and execution [28-30]. Therefore, it is conceivable that ethyl loflazepate has less effect on P300-related information processing, although the subjects did not exhibit any harmful effects on motor skills, visuomotor tracking speed, and delayed memory in the neuropsychological tests.

Secondly, subchronic treatment with drugs produced prolongation in P300 latency only in the Fz regions. Weak prolongation in P300 latency was seen in the etizolam-treated subjects (although this was not statistically significant). The magnitude of prolongation by subchronic ethizolam treatment was reduced when compared to the acute administration of etizolam. In support of this finding, previous studies reported that people develop tolerance to the sedative and cognitive effects of benzodiazepines after subchronic treatments [1]. Interestingly, subchronic treatment of ethyl loflazepate did not prolong the latency in spite of its long elimination rate.

Finally, acute but not subchronic treatment with benzodiazepine reduced P300 amplitude. Based on the magnitude of changes, the main effects on the reduction of P300 amplitude were produced by etizolam (2 mg). This result replicated previous studies that benzodiazepine anxiolytic drugs (lorazepam, clonazepam and alprazolam) induced reductions in P300 amplitude [15,17]. Recent studies demonstrated that reduction in auditory P300 amplitude correlated with the severity of thought disorders [31,32]. Previous studies reported that a single administration of a benzodiazepine drug produced impairment of learning and memory [1-3]. However, the present study showed no aversive effects of the examined drugs on neuropsychological tasks such as attention-needed tasks (trails making test, digit span) and memory (verbal paired associates, digit symbol). Since the subjects were free from abnormal pathological process, alterations in the P300 may be induced by etizolam, not by symptom alleviation due to etizolam.

Differences in the effects on P300 latency between etizolam and ethyl loflazepate could be attributed to their pharmacological properties, such as sedative effects, and affinities for ω-1 and ω-2 sites. Etizolam is short acting (EH of 6 h) whereas ethyl loflazepate is long acting (EH of 122 h).

With regard to limitations of the present study, the sample size was small.

## Conclusions

Acute administration of etizolam induced significant prolongation in P300 latency whereas low dose ethyl loflazepate induced fewer effects on P300 latency in the Fz, Cz and Pz regions than low-dose etizolam. For a while, subchronic administration of etizolam, but not ethyl loflazepate, caused weak prolongation in P300 latency in the Fz but not Cz and Pz regions. In contrast, acute and chronic administrations of etizolam and ethyl loflazepate showed no deficits in motor skills, visuomotor tracking speed, and delayed memory on neuropsychological testing.

## Competing interests

The authors declare that they have no competing interests.

## Authors' contributions

GF conceived the paper, designed the study, performed the psychological measures, collected data, carried out the statistical analysis and drafted the paper; THash performed the psychological measures; YS carried out the statistical analysis and helped draft the study; THase, HW and MF supervised the study; KH and MI designed the study and helped draft the papers. All authors read and approved the final manuscript.
